# Identification of the malonylation modification in *Staphylococcus aureus* and insight into the regulators in biofilm formation

**DOI:** 10.3389/fmicb.2025.1598098

**Published:** 2025-08-19

**Authors:** Xiaoyan Yu, Yi Li, Tingting Yang, Wenjie Li, Xiaozhu Dong, Aixiang Huang, Yanan Shi

**Affiliations:** ^1^College of Food Science and Technology, Yunnan Agricultural University, Kunming, China; ^2^School of Food Science and Engineering, Hainan University, Haikou, China; ^3^Yunnan College of Modern Coffee Industry, Yunnan Agricultural University, Kunming, China

**Keywords:** biofilm formation, lysine malonylation, AgrA protein, *Staphylococcus aureus*, quorum-sensing system

## Abstract

**Background:**

Post-translational modifications (PTMs) are critical regulators of bacterial biofilm formation, but the role of lysine malonylation (Kmal) in biofilm formation is still poorly understood.

**Methods:**

In this study, we analyzed the dynamic changes of protein malonylation of *Staphylococcus aureus* (*S. aureus*) DC15 during biofilm formation based on antibody affinity enrichment combined with quantitative proteomics.

**Results:**

Quantitative profiling identified 2,833 malonylated sites across 788 proteins, with significant enrichment in biofilm-associated proteins. Twelve conserved motifs, including Kmal******R and Kmal****R (* represents any amino acid residue), dominated the malonyl proteome landscape in *S. aureus*. The combined analysis of modified and quantitative proteomics revealed the quorum-sensing system as a key regulatory hub in *S. aureus* biofilm formation. In particular, the response regulator, AgrA, showed decreased expression but increased malonylation at the K2, K11, and K216 sites during *S. aureus* biofilm formation, suggesting functional compensation. Structural and phylogenetic analysis showed that the key malonylation sites (K216) of protein AgrA were evolutionarily conserved in Gram-positive pathogens including Bacillus cereus. Molecular docking analysis found that antimicrobial peptide BCp12 and natural compound chlorogenic acid could bind with the malonylation sites in AgrA (ΔG = −6.888 and −5.302 kcal/mol, respectively).

**Conclusion:**

This study provides a new perspective for understanding the general rules of bacterial biofilm formation and developing broad-spectrum anti-biofilm drugs.

## 1 Introduction

*Staphylococcus aureus* (*S. aureus*) is a commensal of hospital-acquired infections (ESKAPE pathogen) and human skin (Muhammad et al., 2021). Also, *S. aureus* is a global foodborne pathogen that can exist in a planktonic state or as a biofilm. Biofilm formation is an important survival strategy for *S. aureus* to resist host immunity and antibiotic pressure (Farha et al., 2020; Hyerim et al., 2023; Yu et al., 2024), and has important clinical implications. Biofilms can attach to medical devices and host tissue surfaces to form infections that are difficult to clear, such as chronic wound infections, osteomyelitis and endocarditis (Peng et al., 2022). Dormant cells in biofilms can reactivate after treatment, leading to recurrent infections (Carla et al., 2018). Treatment of such infections usually requires surgical removal of the device or excision of the infected tissue (Zohra et al., 2018). Biofilms provide physical barriers and metabolic synergies for bacteria by forming complex multicellular structures, significantly enhancing their pathogenicity and drug resistance (Hyerim et al., 2023; Ruba et al., 2022; Wang et al., 2023). In addition to exhibiting a strong biofilm formation ability, *S. aureus* DC15 is resistant to a variety of antibiotics, including penicillin, oxacillin, and cefoxitin (Adhita et al., 2021). Consequently, traditional antibiotic treatments are ineffective against *S. aureus* DC15, making it a serious threat to food safety and public health.

Biofilm formation initiates with the attachment of planktonic microorganisms to both biotic and abiotic surfaces, culminating in the active or passive diffusion of single cells or cell clusters into mature biofilms (Raimo et al., 2019). Numerous adhesion factors (Xu et al., 2021), regulatory networks, and the synthesis of extracellular matrix components (Uma et al., 2021) play crucial roles in modulating the entire formation process. Recently, the intricate relationship between post-translational modification (PTM) and biofilm formation has garnered significant attention. Research indicates that protein phosphorylation, acetylation, succinylation, and glycosylation are prevalent types of PTM in bacteria (Li Z. et al., 2022). Notably, phosphorylation, acetylation, and succinylation have been identified as closely linked to quorum sensing and biofilm formation in bacterial species. Yao et al. (2019) found that lysine succinylation modification of LuxS at K23 and K30 sites of *Aeromonas hydrophila* can positively regulate the synthesis of AI-2 and promote biofilm formation. Reactive oxygen species produced by the nanomaterial nTiO2 under UV irradiation cause adverse biological effects on various organisms, including cell wall damage, DNA damage, genotoxicity, and disruption of bacterial biofilm formation. nTiO2-induced adaptive mechanisms of biofilm formation were investigated by Zhu et al. (2022) by means of proteomic and phosphorylated proteomic analyses. The results showed that nTiO2 treatment of *Escherichia coli* (*E.coli*) significantly up-regulated iron acquisition and regulated the phosphorylation status of proteins associated with transcription and translation as well as biofilm formation. The bacteria increased iron carrier and extracellular polysaccharide content (by about 57 and 231%, respectively) and enhanced resistance to transcriptionally inhibitory antibiotics. This effectively retarded biofilm development in bacteria exposed to nTiO2. Liu et al. (2023) investigated the regulatory mechanism of H-NS protein acetylation on *Shewanella* biofilm development. It was found that acetylation of H-NS at K19 site inhibited biofilm formation by down-regulating the expression of glutamine synthetase. Glucosyltransferase can regulate the biofilm formation and virulence of *Streptococcus mutans* through lysine acetylation modification (Ma et al., 2021). Alicyn Reverdy et al. constructed single and double mutants of related enzyme genes, and found that some mutants had defects in colony movement or biofilm phenotype changes. After site-directed mutagenesis of the key acetylated proteins YmcA and GtaB, it was found that YmcA (K64R), GtaB (K89R), and GtaB (K191R) mutants showed severe biofilm defects, indicating that protein lysine acetylation plays a global regulatory role in the multicellularity of *Bacillus subtilis*. Thus, we hypothesized that Kmal may be involved in the regulation of proteins associated with biofilm formation in *S. aureus*. The discovery of new functional regulatory proteins, sites, and pathways is important for designing effective inhibitors of biofilm.

Lysine residues in proteins can undergo a variety of PTMs, such as acetylation, crotonylation and 2-hydroxyisobutyrylation, indicating a complex regulatory mechanism of protein function (Hirschey and Zhao, 2015; Huang et al., 2020; Li Z. et al., 2022). The dynamic lysine malonylation/demalonylation of proteins is conserved in most eukaryotes and prokaryotes (Wang et al., 2017). Lysine malonylation (Kmal) regulates protein folding, signaling and energy metabolism by changing the charge of lysine residues (from + 1 to –1 at physiological pH) (Hirschey and Zhao, 2015; Liao et al., 2023). Several studies have implicated malonylation in the pathogenesis of mammalian diseases (Du et al., 2015; Wu et al., 2022). In addition, the role of malonylation modifications in the regulation of foodborne pathogens has received increasing attention. Qian et al. (2016) comprehensively characterized lysine malonylation substrates in *E. coli* found that malonylation modifications were significantly enriched in protein translation, energy metabolism and fatty acid biosynthesis. Malonylation modifications are closely associated with fatty acid metabolism in *E. coli* and also affect citrate synthase activity. Shi et al. (2022) identified differentially expressed malonylated proteins in *S. aureus* after BCp12 treatment. Combined with metabolomics data, the differential proteins and metabolites were found to be significantly enriched in the arginine synthesis pathway, providing new insights into the molecular mechanism of BCp12 inhibition in *S. aureus.*

Previous studies have found that malonylation is a highly prevalent and essential PTM in *S. aureus* DC15. Qualitative proteomic analysis revealed that more than 50% of the proteins in *S. aureus* DC15 contained multiple malonylation sites (Shi et al., 2022). Western blot analysis using pan-malonylated antibodies showed that *S. aureus* DC15 has significantly elevated levels of malonylation during biofilm formation, suggesting its crucial role in population sensing. However, the key downstream malonylation proteins, sites, and pathways of *S. aureus* DC15 quorum sensing, which are crucial for biofilm formation, have not been studied in depth.

By correlating and analyzing modificaomics and proteomics data, it is possible to precisely identify the key proteins that dominate the formation of the biofilm and to reveal the molecular mechanisms underlying their functional changes. This study aimed to (i) quantify the dynamic changes in protein malonylation between the planktonic and biofilm growth phases of *S. aureus* using affinity enrichment and mass spectrometry (MS)-based quantitative proteomics and (ii) identify the malonylated proteins, sites, and pathways that are enriched in the *S. aureus* biofilm. This research provides new insights into the functional regulatory proteins and mechanisms involved in *S. aureus* biofilm formation, which can be used as targets for inhibiting biofilm formation.

## 2 Materials and methods

### 2.1 Bacterial strain and chemical reagents

The *S. aureus* DC15 strain was sourced from the Yunnan Provincial Centre for Disease Control and Prevention (Kunming, China). Bacteria were inoculated in Luria-Bertani (LB) (Guangdong Huankai Microbiology Technology Co., Ltd., Guangzhou, China) and cultured at 37°C for 14 h to obtain log-phase cells for biofilm culture. The bacterial suspension was diluted with phosphate buffered saline (PBS, pH 7.4) to 106 CFU/mL and set aside. The anti-malonyl lysine antibody (lot number PTM-902) and antimalonyllysine beads (lot number PTM-904) were purchased from PTM Biolabs (Hangzhou, China). The chemicals used in this research project are of generic reagent grade or better.

### 2.2 *S. aureus* DC15 biofilm formation

*S. aureus* DC15 biofilm growth was monitored by crystal violet staining at various time intervals over 96 h. Briefly, 2 mL of diluted logarithmic *S. aureus* cells (10^6^ CFU/mL) was added to a 6-well plate containing a sterile cell-attached slide (biosharp, Hefie, China) (biofilm growth carrier) and incubated at 37°C for 4 d. LB medium was used as a blank control. The culture medium was replaced on alternate days. The culture was terminated at 0, 4, 12, 24, 36, 48, 60, 72, and 96 h. The cell-attached slide were retrieved and rinsed with sterile PBS to remove the planktonic bacteria from the surface. Thereafter, the slides were fixed using 2 mL of 2.5% glutaraldehyde solution and then stained with crystal violet solution for 15 min at room temperature. The slides were then washed to remove the excess staining solution, dried, and observed under a light microscope (Carl Zeiss AG, Oberkochen, Germany).

### 2.3 Quantification of *S. aureus* DC15 biofilm

Crystal violet staining combined with microplate reader was used to quantify the biofilm by absorbance measurement (Srdjan et al., 2000). The logarithmically growing *S. aureus* cells were diluted to 10^6^ CFU/mL, and 200 μL of diluted bacterial solution was added to the 96-well plate. The LB culture medium was used as the blank control, and the static culture at 37°C for 4 d. The culture was terminated at 0, 4, 12, 24, 36, 48, 60, 72, and 96 h, respectively. The surface was rinsed with sterile PBS buffer solution to remove the planktonic bacteria, and the residual biofilm was dried. Subsequently, 150 μL of methanol was added to the pore to immobilize the biofilm. After 15 min, the methanol was removed and the biofilm was dried. The biofilm was then dyed with 150 μL of a 0.1% crystal violet solution for 15 min, after which the excess dye solution was rinsed off. Then, the biofilm was dissolved in 150 μL 95% ethanol. The absorbance at 590 nm was measured using a microplate reader (Biotek, Winooski, VT, United States).

### 2.4 Western blotting

Total bacterial proteins from *S. aureus* DC15 biofilms were extracted using a Gram-positive bacterial protein extraction kit (Beyotime, Shanghai, China). Subsequently, 20 μg of the total protein samples were separated by 12% sodium dodecyl sulfate-polyacrylamide gel electrophoresis (SDS-PAGE) and transferred to a polyvinylidene difluoride membrane by electroblotting. The membrane was blocked with 5% bovine serum albumin in Tris-buffered saline (TBS) buffer (25 mmol/L Tris-HCl, pH 8.0; 150 mmol/L NaCl) for 2 h at room temperature. The membrane was then incubated overnight with pan anti-malonyl lysine antibodies (catalog no. PTM-902, Lot: 23056103HA07, PTM Biolabs, Hangzhou, China; dilution, 1:1,000) at 4°C. After washing thrice with TBS with 0.1% Tween20 (TBST) buffer, the membranes were incubated with goat-anti-mouse IgG secondary antibodies (1:5,000; Thermo Fisher Scientific, Waltham, MA, Unites States) for 1 h at 37°C. Protein bands from SDS-PAGE and Western blotting were visualized using Coomassie Brilliant Blue dye and ECL substrate kits, respectively.

### 2.5 Lysine malonylation modification analysis

#### 2.5.1 Protein extraction

Bacterial cultures were mixed with lysis buffer containing 8 M urea, 1% protease inhibitor cocktail (Sigma-Aldrich, Darmstadt, Germany), 3 μM trichostatin A (TSA), 50 mM nicotinamide (NAM), and 1% phosphatase inhibitor (Sigma-Aldrich, Darmstadt, Germany). The mixture was sonicated three times on ice using a high-intensity ultrasonic processor (Scientz, Ningbo, China) and centrifuged at 12,000* g* for 10 min at 4°C. The supernatant was collected and the protein concentration was determined using a bicinchoninic acid (BCA) (Beyotime, Shanghai, China) kit, according to the manufacturer’s instructions.

#### 2.5.2 Trypsin digestion

Proteins were isolated by gradually adding samples to trichloroacetic acid (TCA) (final concentration of 20% v/v). The protein mixture was vortexed, incubated at 4°C for 2 h, and centrifuged at 4,500 *g* for 5 min at 4°C. The protein precipitate was washed thrice with precooled acetone and dried for 2 h. Thereafter, the protein sample was resuspended in 100 mM triethylammonium bicarbonate buffer (TEAB) and ultrasonically dispersed. The protein sample was digested overnight with trypsin (50:1 protein:trypsin ratio) and then treated with 5 mM dithiothreitol for 60 min at 37°C and 11 mM iodoacetamide for 45 min at room temperature. Finally, the peptides were desalted by a C18 solid phase extraction column.

#### 2.5.3 Pan-antibody-based PTM enrichment

To enrich Kmal-modified peptides, the digested peptides were dissolved in nuclear and cytoplasmic extraction (NETN) buffer (100 mM NaCl, 1 mM ethylenediaminetetraacetic acid (EDTA), 50 mM Tris-HCl, and 0.5% NP-40, pH 8.0) and incubated overnight with pre-washed anti-malonyl lysine beads (Catalog no. PTM-904; lot: TAJB09B02; PTM Biolab, Hangzhou, China) at 4°C with gentle shaking. The beads were then washed four times with NETN buffer and two times with water to remove the unbound peptides. The bound peptides were eluted from the beads using 0.1% trifluoroacetic acid. Finally, the eluted fractions were combined and vacuum-dried. For liquid chromatography-tandem mass spectrometry (LC-MS/MS) analysis, the resulting peptides were desalted using C18 ZipTips (Millipore, Billerica, United States) according to the manufacturer’s instructions.

#### 2.5.4 LC-MS/MS analysis

The 5–10 μg digested peptides were dissolved in solvent A (0.1% formic acid and 2% acetonitrile in water) and loaded onto a homemade reverse-phase analytical column (25 cm length, 75/100 μm i.d.). The peptide components were then separated using the following protocol: 6−24% solvent B gradient (0.1% formic acid in acetonitrile) over 70 min, 24−35% solvent B gradient over 14 min, 35−80% solvent B gradient over 3 min, and 80% solvent B held for 3 min. All the samples were analyzed at a constant flow rate of 450 nL/min on a nanoElute ultra-high performance liquid chromatography (UHPLC) system (Bruker Daltonics, Billerica, United States). The peptides were subjected to capillary source followed by the timsTOF Pro (Bruker Daltonics, Billerica, United States) mass spectrometry. The applied electrospray voltage was 1.60 kV. Precursors and fragments were analyzed at the TOF detector, with an MS/MS scan range from 100 to 1,700 m/z. The timsTOF Pro was operated in the parallel accumulation−serial fragmentation (PASEF) mode. Precursors with charge states 0−5 were selected for fragmentation, and 10 PASEF-MS/MS scans were acquired per cycle. The dynamic exclusion was set to 30 s.

#### 2.5.5 Database search and data processing

The resulting MS/MS data were processed using the MaxQuant search engine (v.1.6.15.0). Tandem mass spectra were searched against the UniProt *S. aureus* taxonomy database concatenated with a reverse decoy database. Trypsin/P was specified as the cleavage enzyme (allowing up to two missing cleavages). The mass tolerance for precursor ions was set at 40 ppm in the first and main searches. The mass tolerance for fragment ions was set at 0.04 Da. Carbamidomethyl (C) was specified as the fixed modification and variable modifications were oxidation (M), and malonylation (K). False discovery rates (FDR) at protein, peptide and modification level were all set as 1%.

### 2.6 Quantitative proteomics

#### 2.6.1 Protein extraction

Logarithmic phase planktonic cells and 96 h biofilm cultures of *S. aureus* DC15 were collected and centrifuged at 6,000* g* for 15 min at 4°C. Protein extraction was conducted according to the method described by Shi et al. (2021), with slight modifications. The bacterial samples were flash-frozen with liquid nitrogen and ground to a powder. Subsequently, two sets of samples were treated with lysis buffer (1% SDS, 1% protease inhibitor, 1% phosphatase inhibitor, 50 μM PR-619, 3 μM TSA, 50 mM NAM) and ultrasonically lysed. The samples were then centrifuged at 12,000* g* for 10 min at 4°C and the supernatant containing the total protein was collected. The protein concentration was determined using the BCA protein assay kit (Beyotime, Shanghai, China).

#### 2.6.2 Trypsin digestion

For digestion, the extracted protein sample was reduced with 5 mM dithiothreitol at 56°C for 30 min and alkylated with 11 mM iodoacetamide for 15 min at room temperature in the dark. The reduced protein was diluted with 100 mM TEAB containing < 2 M urea. Thereafter, the protein samples were subjected to two rounds of trypsin digestion: Round 1 involving overnight digestion at 1:50 trypsin to protein mass ratio and round 2 involving 4 h digestion at 1:100 trypsin to protein mass ratio. Finally, the peptides were desalted using the C18 solid phase extraction column.

#### 2.6.3 LC-MS/MS identification

The tryptic peptides were dissolved in solvent A, directly loaded onto a home-made reversed-phase analytical column (25 cm length, 100 μm i.d.). The mobile phase consisted of solvent A (0.1% formic acid, 2% acetonitrile/in water) and solvent B (0.1% formic acid in acetonitrile). Peptides were separated with following gradient: 0–8 min, 9–24% B; 8–12 min, 24–35% B; 12–16 min, 35–80% B; 16–20 min, 80% B, and all at a constant flow rate of 500 nl/minon a NanoElute UHPLC system (Bruker Daltonics). The peptides were subjected to capillary source followed by the timsTOF Pro 2 mass spectrometry. The electrospray voltage applied was 1.75 kV. Precursors and fragments were analyzed at the TOF detector. The timsTOF Pro was operated in data independent parallel accumulation serial fragmentation (dia-PASEF) mode. The full MS scan was set as 300–1,500 (MS/MS scan range) and 20PASEF (MS/MS mode) -MS/MS scans were acquired per cycle. The MS/MS scan range was set as 400–850 and isolation window was set as7 m/z.

#### 2.6.4 Database search

In this study, protein relative quantification was performed by spectral counting. The relative quantification of proteins was achieved by comparing the number of MS/MS identification spectra from the same protein in multiple LC-MS/MS data sets (Zhu et al., 2010). The DDA data were processed using Spectronaut (v.17.0) software coupped with Pulsar search engine. Tandem mass spectra were searched against *Staphylococcus aureus*_subsp.aureus71193_1155084_UP_20221227_seqkit. fasta (80,424 entries) concatenated with reverse decoy database. The max missing cleavages was set as 2. Carbamidomethyl on Cys was specified as fixed modification. Acetylation on protein N-terminal, oxidation on Met were specified as variable modifications. False discovery rate (FDR) of protein, peptide and PSM was adjusted to < 1%. The corresponding spectral library was imported into Spectronaut (v.17.0) software to predicts the retention time by non-linear correction and searched against with DIA data. Enrichment pathway analysis was performed using the Kyoto Encyclopedia of Genes and Genomes (KEGG) database.

### 2.7 Bioinformatics analysis

Subcellular localization of all the malonylated proteins from the three experiments was predicted using WOLF PSORT. The sequence models for motif analysis were composed of malonyl-21-mers, with 10 amino acids upstream of the Kmal site and 10 amino acids downstream of the Kmal site. Kmal motifs were then predicted using the MoMo software (motif-x algorithm). Based on the results of MoMo analysis, the degree of change scoring (DS) of the frequency of amino acid occurrences near the modification site was presented in the form of a heat map. DS was calculated using the following formula: *DS* = -Log10(p-value)*sign(diff.percent). S. aureus protein sequences from the UniProt database were used as the background database parameter, and other parameters were set to default. *S. aureus* genomic information was used as background, and *P* < 0.05 were used as thresholds for differentially expressed proteins (DEPs) and differentially malonylated proteins (DMPs). Gene ontology (GO) and domain annotation of DMPs were performed using DAVID 6.7. KEGG^1^ and MetaboAnalyst,^2^ were used for pathway enrichment analysis of DMPs.

### 2.8 Molecular docking analysis

The crystal structure of AgrA was obtained from PDB (PDB ID: 4XQQ).^3^ The structure of the docked peptide, BCp12, was constructed using PyMOL 2.5.5 and then imported into Chem 3D software for optimization. The 3D structure of the small molecule chlorogenic acid (CGA) was obtained from the PubChem database and was energy minimized under the MMFF94 force field.

Molecular docking was performed using AutoDock Vina v.1.2.3 software, after removing the water molecules, salt ions, and small molecules from the receptor proteins using PyMol v.2.5.5. All processed small molecules and receptor proteins were converted into the PDBQT format using ADFRsuite v.1.03. The docking results were visualized and analyzed using PyMol v.2.5.5.

### 2.9 Statistical analysis

Three independent experiments were performed for each experiment. Statistical analyses such as independent samples *t*-test and ANOVA were performed in IBM SPSS Statistic 20.0 software. Data are expressed as mean ± standard deviation (SD), *P* < 0.05 was considered a significant difference. GraphPad Prism 5 was used for graphical evaluation.

## 3 Results and Discussion

### 3.1 *S. aureus* DC15 biofilm analysis

**FIGURE 1:**
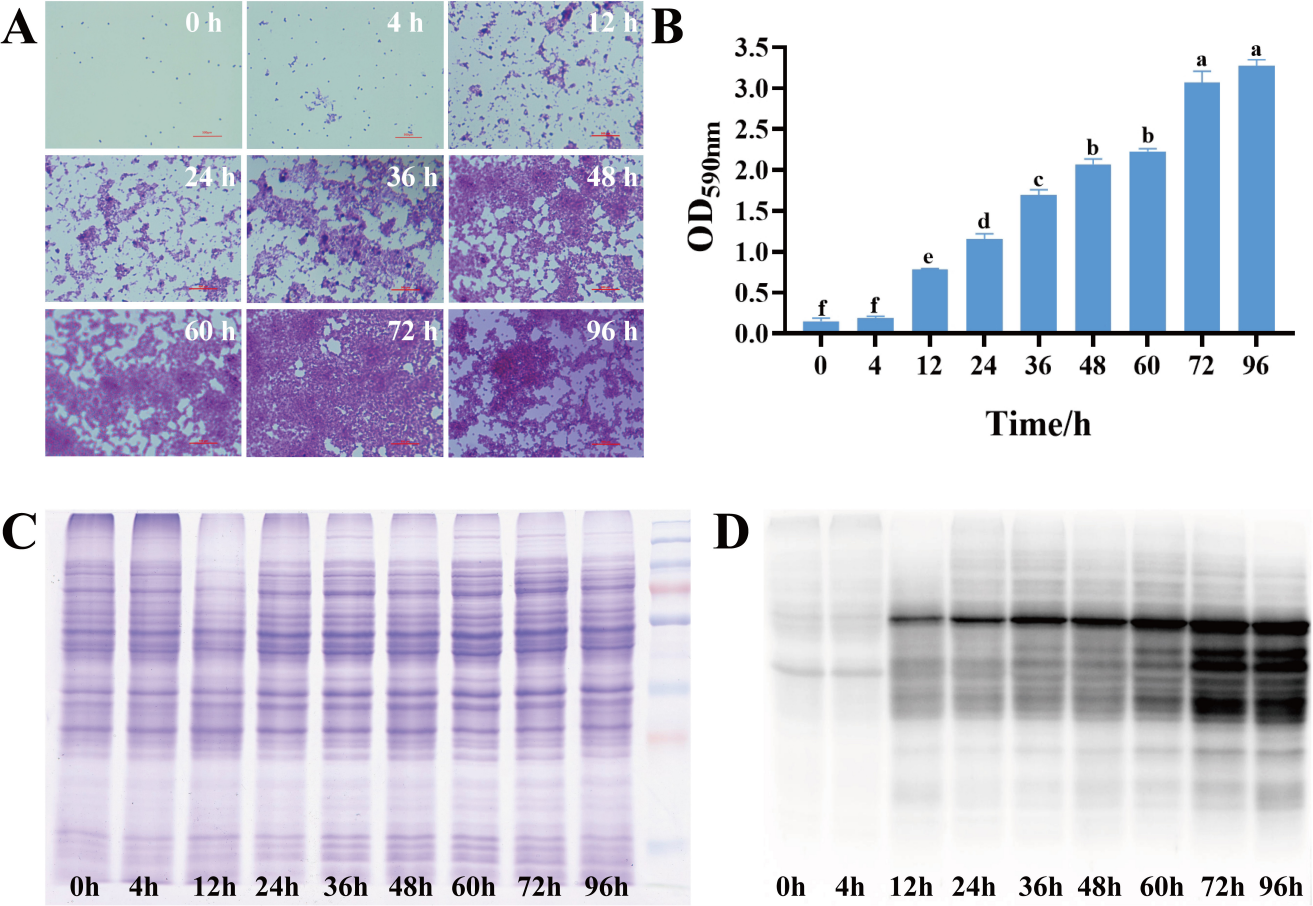
**(A)** Dynamic changes in *S. aureus* biofilm (0–96 h); **(B)** Quantification of *S. aureus* biofilm (0–96 h) (different lowercase letters on the column indicate that the difference is statistically significant, *P* < 0.05); **(C)** SDS-PAGE of total proteins in different growth stages of *S. aureus*; **(D)** Western blot of malonylated proteins in different growth stages of *S. aureus*.

To investigate the dynamics of *S. aureus* biofilm growth, *S. aureus* DC15 cultures were cultured for 0–96 h to allow biofilm formation, and the biofilms were detected at different time points by crystal violet staining (Figure 1A). Light microscopic analysis revealed that *S. aureus* DC15 cells were in a free state from 0 to 4 h of incubation but showed partial aggregation and adhesion after 12 h of incubation. A previous study found that *Acinetobacter baumannii* cells formed a reticulum (the initial state of a biofilm) after 24 h of incubation. As time increased, the bacteria formed a thick biofilm (Pan et al., 2024). Another study showed that the complex three-dimensional structure of the biofilm was fully formed and the bacterial population within the biofilm was in an active state after 48 h of incubation in *Salmonella* (Turki et al., 2012; Wang H. et al., 2013). Li Y. et al. (2022) observed *S. aureus* DC15 by SEM and CLSM after 48 h of culture. The results showed that the bacteria were connected to each other and formed cell clusters through the extracellular matrix, and the membrane contained a large number of live bacteria. Similarly, in this study, *S. aureus* DC15 formed a reticular structure after 24 h of incubation. After 48 h of incubation, the cells aggregated and adhered to the carrier surface, forming a large membrane structure that surrounded the entire field of view. After 96 h of incubation, *S. aureus* DC15 formed a mature biofilm. The amount of biofilm formation was quantified by measuring the absorbance value after crystal violet staining (Figure 1B). The results showed that the amount of biofilm formation of *S. aureus* increased with time. The amount of biofilm formation reached the maximum after 72 h and 96 h of culture, indicating that the biofilm state was stable at this time, which further verified the results of optical microscope observation.

### 3.2 Malonylation status in planktonic and biofilm cells of *S. aureus* DC15

Malonylated proteins have been reported to be involved in a variety of biological processes in *S. aureus* (Shi et al., 2022; Shi et al., 2021). To investigate the effect of protein malonylation status on the biofilm formation ability of *S. aureus* DC15, we compared the malonylation levels in planktonic and biofilm cells. For this, total proteins were isolated from the planktonic cells and biofilm of *S. aureus* DC15 and analyzed by Western blotting using pan anti-malonyl lysine antibodies. The results showed that the protein malonylation levels changed significantly, while protein expression levels were within the normal range and remained unchanged during *S. aureus* DC15 biofilm formation (Figure 1C). Li Z. et al. (2022) showed that malonylation levels were significantly higher in *Streptococcus mutans* in the biofilm state compared to the planktonic state, suggesting that Kmal plays an important role in regulating the physiological activities of cariogenic biofilms. Consistently, the present study found that biofilm growth significantly up-regulated the overall malonylation levels in *S. aureus* DC15 (Figure 1D), further emphasizing the potential importance of malonylation on biofilm formation. To investigate the effect of normal malonylation status on biofilm formation, this study quantified malonylation in planktonic and biofilm cells of *S. aureus* DC15 using quantitative proteomics. This approach allowed us to reveal the dynamic changes and functional significance of malonylation in biofilm formation. It is worth noting that the changes in malonylation levels observed during biofilm maturation may not be exclusively driven by biofilm-specific factors, but may also be influenced by the general factor of bacterial aging in general. Bacterial cells undergo intrinsic age-related changes during growth, whether in a biofilm environment or in a planktonic state, and these changes may have an impact on malonylation levels. Therefore, the potential contribution of bacterial aging in general to malonylation levels should be fully considered as a possible confounding or parallel influence in future studies. In-depth analyses of the relative contributions of biofilm-specific factors and general bacterial aging factors in changes in malonylation levels.

### 3.3 Malonylation proteomics to identify DMPs

**FIGURE 2:**
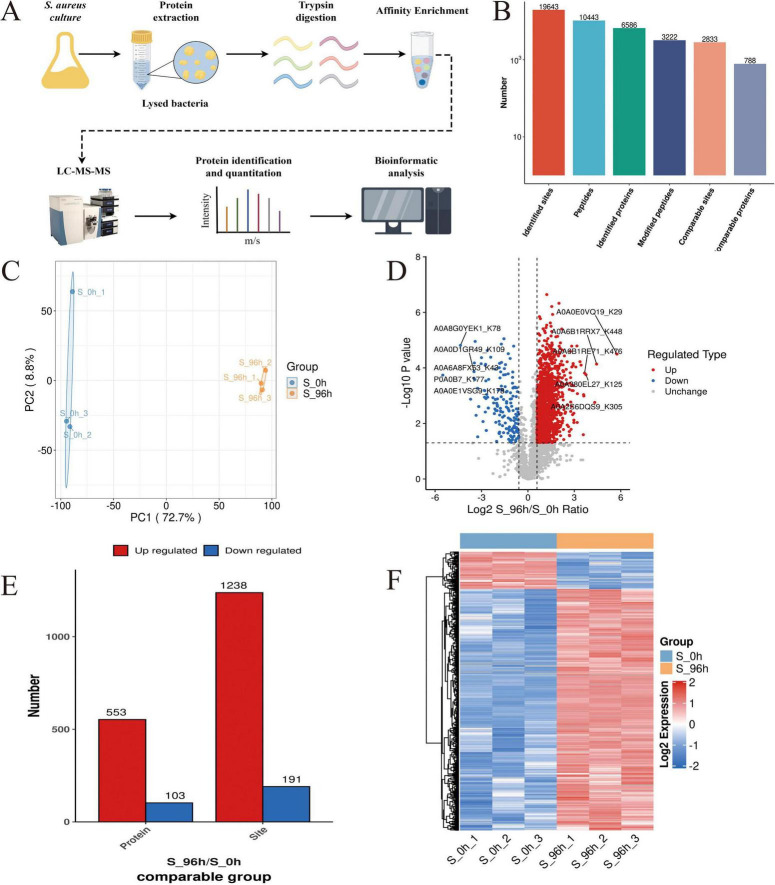
*S. aureus* Kmal modification omics analysis (*n=3*). **(A)** The workflow of *S. aureus* Kmal modification omics; **(B)** Identification of malonylated proteins and Kmal sites in *S. aureus*; **(C)** Principal component analysis of malonylated proteins in *S. aureus* planktonic cells (0 h) and biofilm cells (96 h); and **(D)** Volcano map of differential modification sites of planktonic bacteria and biofilm of *S. aureus*; **(E)** Differentially expressed malonylated proteins and sites; **(F)** Hierarchical cluster analysis of DMPs in *S. aureus* planktonic cells (0 h) and biofilm cells (96 h) (*P* < 0.05).

Using a specific anti-malonyl-lysine pan-antibody for enrichment, coupled with proteome analysis (Figure 2A). A total of 3,222 modified peptides were identified in this study, and the matching results resolved 19,643 modification sites. 2,833 malonylated sites were quantified in 788 quantified malonylated proteins with a confidence level of > 0.75 (Figure 2B). PCA plots showed significant differences between the planktonic cell group and the biofilm group, indicating significant changes in the malonylation state of proteins during *S. aureus* DC15 biofilm formation (Figure 2C). The up-regulation and down-regulation of differential modification sites were visualized by volcanic maps (Figure 2D). A total of 656 DMPs were identified, of which 553 contained 1,238 up-regulated malonylation sites and 103 contained 191 down-regulated malonylation sites (Figure 2E and Supplementary Datasheet 1). These results indicate that protein malonylation status changes significantly during biofilm formation, highlighting its key role in regulating *S. aureus* DC15 biofilm formation as well as in bacterial adaptation and pathogenicity.

To determine the amino acid composition near the Kmal sites, we used intensity maps (Supplementary Figure S1) to systematically detect the amino acid sequences on both sides of the Kmal site. The different preferences for amino acids reflect the specific recognition sites of enzymes that catalyze malonylation at different stages (Xie et al., 2016). By constructing sequence markers, we quantitatively analyzed the enrichment or deletion of amino acids at various positions around the Kmal site, thereby characterizing the potential sequence signature pattern near the Kmal residues in *S. aureus* DC15. The results showed that there were 12 significantly conserved sequence motifs, including Kmal******R, Kmal****R, Kmal*****R, Kmal******K, Kmal*******R, Kmal********R, Kmal*******K, Kmal********K, Kmal*****K, Kmal*********R, Kmal*********K, and Kmal***R (* represents any amino acid residue), near the Kmal site (Supplementary Figure S2). There are significant differences in the motifs of different acylation modifications (Lv et al., 2021). For example, the acetylation sites are usually surrounded by hydrophobic amino acids such as phenylalanine, tyrosine and negatively charged aspartic acid. Succinylation enriched hydrophobic valine and glycine at the –1 and + 1 positions of lysine residues. This difference may imply that different acylation modifications are catalyzed by different acyltransferases, or that the same type of acyltransferases have different substrate specificity preferences.

To further resolve the expression patterns of DMPs, we performed hierarchical cluster analysis, which showed that the three independent replicate experiments showed a high degree of consistency (Figure 2F). The DMP sites could be divided into two clustering groups: 0 h and 96 h groups (Figure 2F). DMPs in the 0 h group showed a down-regulation in malonylation intensity, while DMPs in the 96 h group showed an upregulation in the malonylation intensity after biofilm formation. This complementary pattern suggests a close association between malonylation and biofilm formation. Notably, this study revealed a selective preference for specific amino acids in the vicinity of the Kmal site, indicating their potential as enzyme recognition sites for malonylation. The identification of 12 conserved motifs further suggests that malonylation in *S. aureus* DC15 may be subjected to precise targeted regulation.

### 3.4 Quantitative proteomics analysis of DMPs in *S. aureus* DC15

To determine the molecular mechanisms underlying the regulatory role of protein malonylation in *S. aureus* DC15 biofilm formation, we integrated malonylation and quantitative proteomics analyses. We systematically identified the target malonylation proteins and the associated signaling pathways that play key regulatory roles in *S. aureus* DC15 biofilm formation. This integrated multi-omics analysis elucidated the dynamics of protein expression and revealed the important regulatory role of PTMs during biofilm formation, thus deepening our understanding of the molecular regulatory network of *S. aureus* biofilm formation.

**FIGURE 3:**
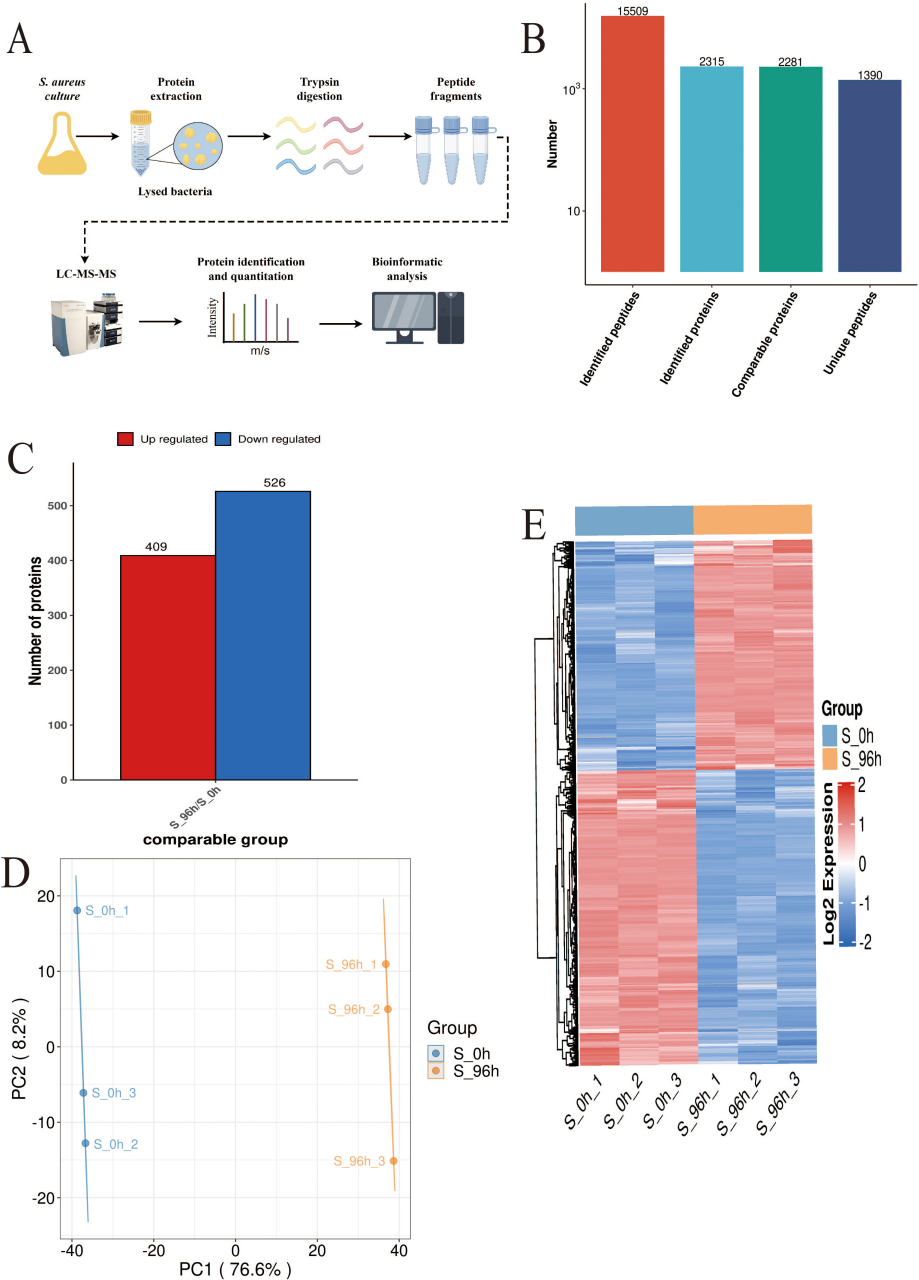
*S. aureus* quantitative proteomics analysis (*n=3*). **(A)** The workflow of *S. aureus* quantitative proteomics; **(B)** The protein quantity in S. aureus planktonic cells (0 h) and biofilm cells (96 h); **(C)** The number of DEPs in the planktonic cells (0 h) and biofilm cells (96 h) of *S. aureus*; and **(D)** Principal component analysis of DEP expression in the planktonic cells (0 h) and biofilm cells (96 h) of *S. aureus* and **(E)** Hierarchical cluster analysis of DEPs in the planktonic cells (0 h) and biofilm cells (96 h) of *S. aureus* (*P* < 0.05).

Proteomics analysis (Figure 3A) identified 2,315 high-confidence proteins in *S. aureus* DC15 cells (Figure 3B). Comparative analysis of *S. aureus* DC15 biofilm and planktonic cells revealed 935 significantly differentially expressed proteins (DEPs), of which 409 were up-regulated and 526 were down-regulated (Figure 3C and Supplementary Datasheet 2). Results of the principal component analysis showed that the protein expression profiles of the 0 and 96 h time points showed a significant separation trend (Figure 3D), and this differential expression pattern was further verified and visualized by hierarchical clustering analysis (Figure 3E).

### 3.5 Comparative analysis of protein modification omics and proteomics

**FIGURE 4:**
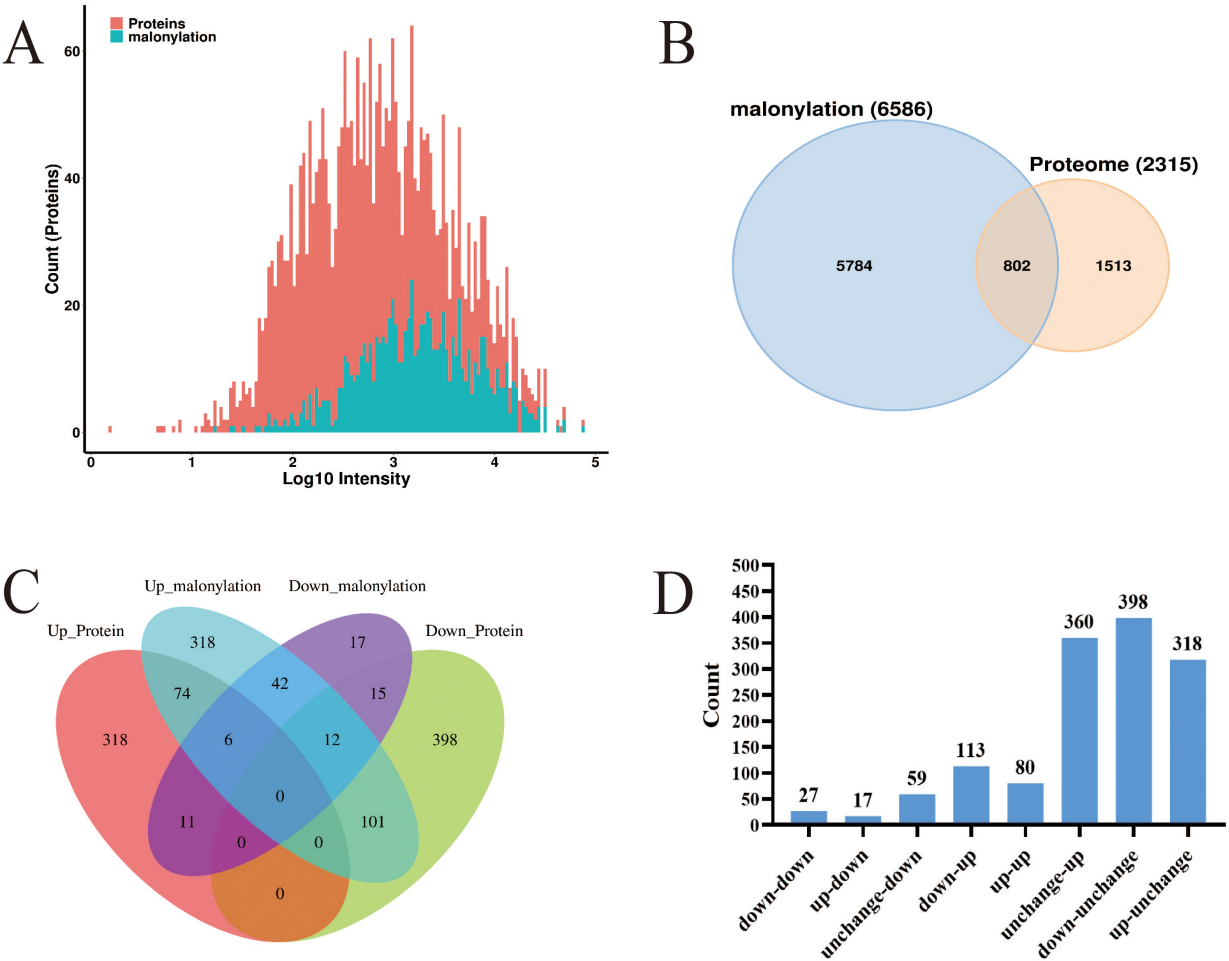
**(A)** Histogram of the protein intensity distribution in modified and proteome groups; red represents total proteins and blue represents modified proteins; **(B)** Wayne diagram of the modified and proteome groups; **(C)** Venn diagram of DEPs in the modified and proteome groups; **(D)** The histogram of differential protein distribution between protein group and modified group.

Through a systematic comparative analysis of proteomics and modified proteomics, we found that the number of total proteins was significantly higher than that of malonylated modified proteins. From Figure 4A, it can be observed that total protein is widely distributed and peaks in the medium intensity range (Log10 intensity is about 2–4), indicating that the intensity of most proteins is concentrated in this range. In contrast, the distribution of malonylated proteins is relatively narrow and the abundance is low, mainly concentrated in the high intensity region (Log10 intensity is about 3–4). The number of total proteins reached a maximum at moderate intensity, while the number of malonylated proteins reached a maximum at higher intensity. As a specific modified protein, the distribution of malonylated protein may be more concentrated in certain intensity ranges. It has been shown that PTM are widely present in eukaryotes but in low overall abundance, which is consistent with the predominance of unmodified proteins observed in this study (Li, 2024; Wang Y. et al., 2013). Mann and Jensen showed that although more than 50% of proteins may undergo PTMs, individual modification types typically cover only 1–10% of the proteome in a given physiological state (Khoury et al., 2011; Xiong et al., 2016). The correlation between proteomics and modificationomics was further revealed by Wayne diagram analysis (Figure 4B). The results revealed that the proteome and malonylation group shared 802 proteins, accounting for 34.6% of the total proteins. These common proteins may play an important role in the regulation of cell function, and their biological functions may be significantly affected by malonylation modification. Venn diagrams (Figure 4C) and histogram (Figure 4D) were constructed to show the changes in protein expression and malonylation in planktonic and biofilm cells of *S. aureus* DC15. The analysis showed that of the 398 proteins, 113 showed down-regulated expression but up-regulated malonylation. This expression-modification reverse regulation pattern is highly similar to the post-translational compensation mechanism observed by Zhao et al. (2022) in *Pseudomonas aeruginosa* biofilms. Their study found that the downregulation of PqsE (group sensing-related protein) expression was accompanied by an upregulation of its succinylation and that this regulatory pattern is involved in *P. aeruginosa* biofilm homeostasis. The direct regulatory relationship between protein expression level and malonylation status during biofilm formation, suggests the presence of a post-translational compensation regulation mechanism in *S. aureus*, which may allow it to adapt to surface attachment growth.

### 3.6 Quorum sensing system in *S. aureus* DC15 biofilm formation

**FIGURE 5:**
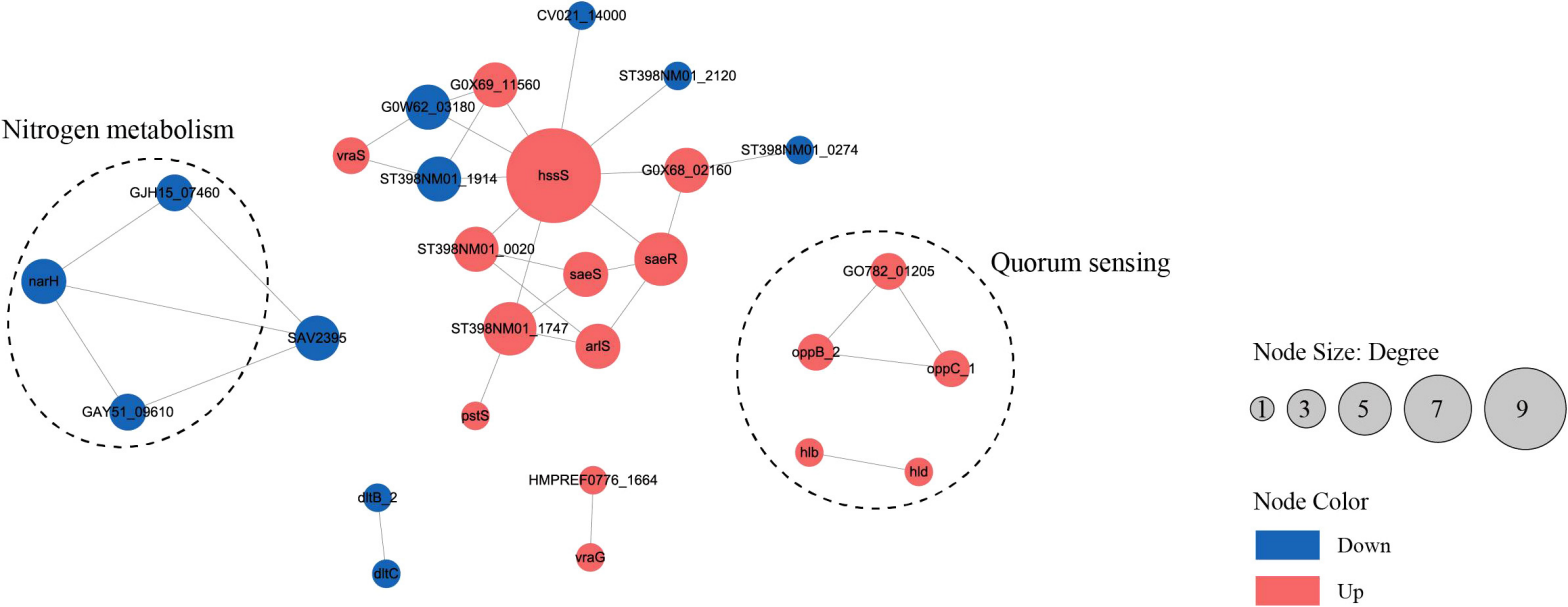
The interaction network of differentially expressed proteins or genes in the planktonic cells (0 h) and biofilm cells (96 h) of *S. aureus*. (*P* < 0.05, red represents up-regulation, blue represents down-regulation).

To analyze the cellular and physiological changes of *S. aureus* during biofilm formation, we performed systematic bioinformatics analyses of the DEPs and DMPs (*P* < 0.05). The key DEP interaction network (Figure 5 and Table 1) showed that DEPs were significantly enriched in the core regulatory pathways of biofilm formation, including quorum sensing, indicating that protein malonylation modification may have a potential role in the biofilm formation of *S. aureus* DC15.

**TABLE 1:** Differentially expressed genes in planktonic cells (0 h) and biofilm cells (96 h) of *S. aureus.*

Number	Gene name	S_96 h/S_0 h Ratio	S_96 h/S_0 h *P*-value	Regulated type
1	*narH*	0.43878263	1.98614E-06	Down
2	*dltC*	0.616784233	0.014010428	Down
3	*dltB_2*	0.140543474	0.026076355	Down
4	SAV2395	0.60980624	0.004143304	Down
5	GAY51_09610	0.515561233	2.8831E-07	Down
6	GJH15-07460	0.327578471	3.12099E-05	Down
7	G0W62_03180	0.476925507	2.82748E-05	Down
8	ST398NM01_1914	0.646929814	0.009740088	Down
9	CV021_14000	0.617708953	0.002860398	Down
10	ST398NM01_2120	0.592645532	0.001258497	Down
11	ST398NM01_0274	0.444512763	0.000525972	Down
12	G0 × 69_11560	1.635860813	0.023515569	Up
13	ST398NM01_0020	1.520582963	0.000180822	Up
14	ST398NM01_1747	1.948683443	3.822E-05	Up
15	HMPREF0776_1664	2.44357838	7.97046E-05	Up
16	G0 × 68_02160	1.558431044	0.003421906	Up
17	GO782_01205	1.598562139	0.000159827	Up
18	*pstS*	6.425917838	1.31584E-05	Up
19	*hssS*	1.550887231	0.025683637	Up
20	*saeS*	1.519690021	0.000201498	Up
21	*arlS*	1.508534129	0.000237811	Up
22	*vraG*	1.879065854	0.001071408	Up
23	*saeR*	1.505556948	0.000162555	Up
24	*oppB_2*	1.705691906	0.033715237	Up
25	*oppC_1*	1.623791042	0.00151971	Up
26	*hlb*	2.183050442	8.10242E-05	Up
27	*hld*	1.533753182	0.000242232	Up
28	*vraS*	1.87409189	0.00011457	Up

Studies have reported that β-hemolysin *(hlb*) and δ-hemolysin (*hld*) enhance the stability of biofilm structure by regulating hemolysin secretion, promoting intercellular aggregation, and extracellular matrix diffusion (Cheung et al., 2014; Saravanan et al., 2012). In addition, *oppB_2* and *oppC_1*, which encode subunits of the oligopeptide permease system (Opp), may activate the accessory gene regulator (Agr) system by translocating auto-inducing peptides, further enhancing the efficiency of population-sensing signaling (Polaske et al., 2023; Thoendel et al., 2010; Wang et al., 2005). In this study, we found that the Agr system and its direct target genes, *hlb, hld*, *oppB_2*, and *oppC_1* were significantly up-regulated in the reciprocal network, suggesting that they play an important role in *S. aureus* DC15 biofilm formation. In addition, these genes are associated with quorum sensing and may be involved in communication and coordination between bacteria, thereby affecting biofilm formation.

The same *saeR* and *hssR* as in Michalik et al. (2017) and Surmann et al. (2014) studies of *S. aureus* transcription factor regulators also appeared in the present study. The sensor kinase (*hssS*) exhibits a high degree of connectivity in an interoperational network with extensive interactions with multiple genes. *hssS* acts as a histidine kinase sensor in a two-component system, and undergoes a conformational change when membrane hemoglobin binds to a response regulator (*hssR*). The enzyme activity is activated and the consequently phosphorylated *hssR* subsequently regulates the expression of a range of genes that are closely linked to bacterial survival, adaptation and pathogenicity (Saillant et al., 2024). It has been shown that *hssS* deficiency leads to reduced survival of Listeria monocytogenes in oxidative stress environments, suggesting that *hssS* plays an important role in bacterial growth as well as biofilm formation (An et al., 2022). Studies have shown that *S. aureus* lacking the *hssS* shows obvious defects in biofilm formation, indicating its key role in biofilm formation. In this study, *hssS* was significantly up-regulated (Figure 5) and showed a high degree of connectivity in the interaction network. These results suggest that *hssS* may serve as a metabolic-signal hub, coordinating the structure and function of *S. aureus* biofilm by integrating its nitrogen metabolism and quorum sensing.

### 3.7 Effect of PTM of AgrA on *S. aureus* DC15 biofilm formation

**FIGURE 6:**
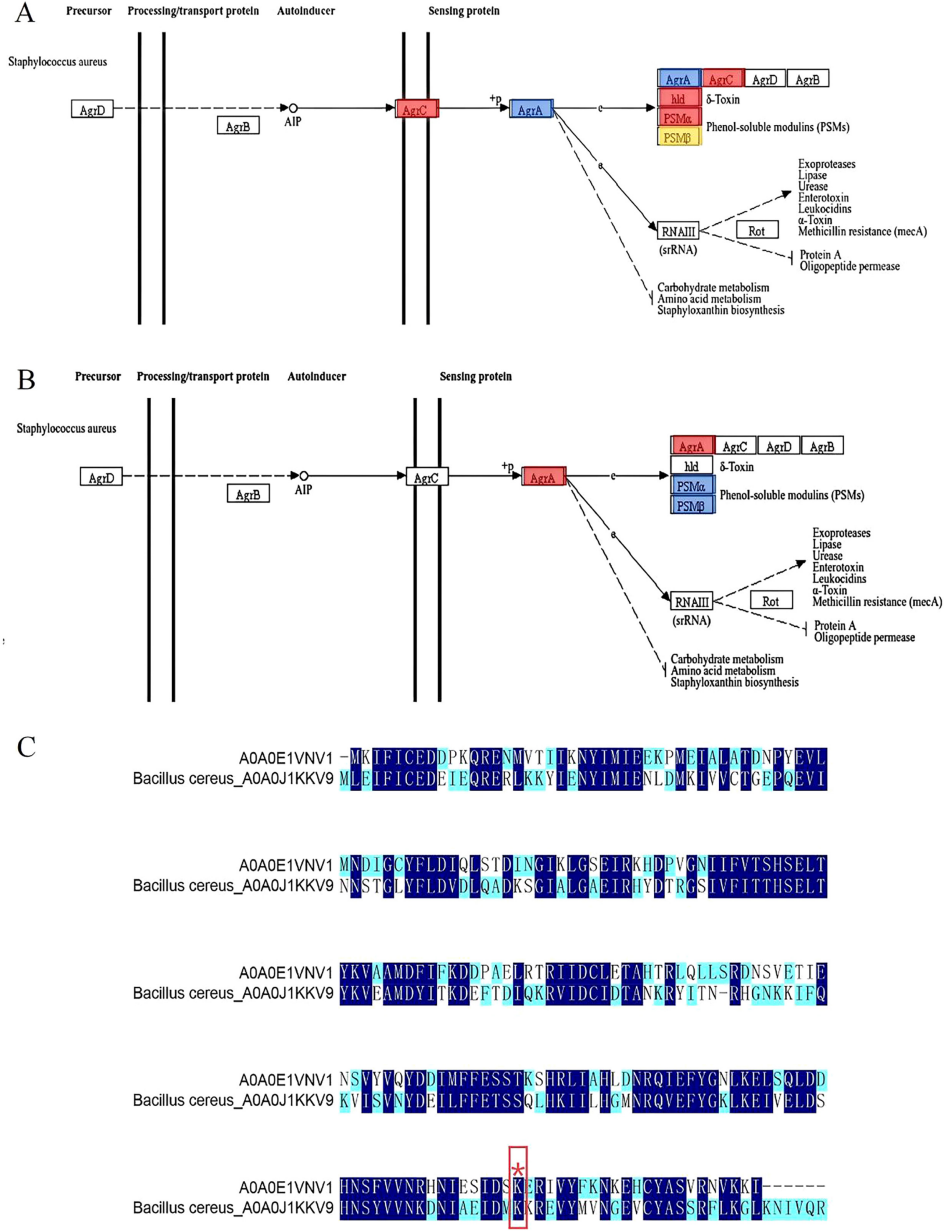
**(A)** Changes in AgrA in the quantitative proteome were blue-labeled and mapped to the KEGG pathway; **(B)** Quantification of AgrA changes in the malonylation-modified proteome was red-labeled and mapped to the KEGG pathway; **(C)** Sequence comparison of *S. aureus*-A0A0E1VNV1 and B. cereus-A0A0J1KKV9. (Different colors indicate the degree of conservation of the locus, with dark blue representing conservation and light blue and white representing incomplete conservation. ‘*’ represents lysine malonylation sites).

Novick and Geisinger (2008) found that the Agr system senses population density through autoinducer peptide and regulates the expression of virulence factors and biofilm inhibitory genes. As a key regulator of the quorum-sensing system, AgrA plays an important role in cell density-dependent communication in *S. aureus* (Li Y. et al., 2022). AgrA was highly expressed in *S. aureus* planktonic cells, which was conducive to the rapid response of bacteria to environmental signals and the initiation of the quorum-sensing process. However, during biofilm formation, the expression of Agr A was significantly down-regulated (Figure 6A and Table 2), but the key malonylation modification site (K2, K11, K216) was significantly up-regulated, and there was no significant difference or change in the modification site of K225 (Figure 6B and Table 3). Yang et al. (2016) found that Agr activity is significantly reduced in *S. aureus* biofilm and may subsequently reduce the release of cytolysin to maintain biofilm structure. Yarwood and Schlievert (Yarwood and Schlievert, 2003) further demonstrated that *agr*-deficient strains show enhanced biofilm formation ability. PTM can affect all aspects of protein function, one of which is the proteolytic stability of proteins. PTMs can occur on specific amino acids in the regulatory domain of the target protein. These regions are called degrons. PTMs can act as signals to accelerate protein degradation, or delay degradation and stabilize proteins. This indicates that PTMs play an important role in regulating protein stability (Lee et al., 2023). Therefore, the down-regulation of AgrA key modification sites may be an active regulatory strategy for biofilm development. Malonylation was found to inhibit the glycolytic enzyme GAPDH. SIRT5 regulates the activity of the glycolytic enzyme GAPDH by de-malonylating a key residue, K184, located at the interface of the glycolytic enzyme homodimer (Nishida et al., 2015). The pattern of inverse regulation of expression and modification suggests that AgrA malonylation may affect its protein stability and thus play a regulatory role in *S. aureus* biofilm formation.

**TABLE 2:** Quantification of AgrA in proteome.

Protein accession	Gene name	S_96 h/S_0 h ratio	S_96 h/S_0 h *p*-value	Regulated type
A0A0E1VNV1	AgrA	0.50443486	0.008445307	Down

**TABLE 3:** The key modification sites of AgrA in malonyl modification group.

Protein accession	Gene name	Position	Amino acid	S_96 h/S_0 h ratio	S_96 h/S_0 h *p*-value	Regulated type
A0A0E1VNV1	AgrA	2	K	2.704059591	0.011677531	Up
A0A0E1VNV1	AgrA	11	K	1.614577152	0.004489273	Up
A0A0E1VNV1	AgrA	216	K	3.662741684	0.037293559	Up
A0A0E1VNV1	AgrA	225	K	1.242217326	0.170342986	None

Shi et al. (2022) found that 31 lysine malonylation sites in *S. aureus* were homologous to lysine malonylation sites previously identified in *E. coli*, indicating that malonylation sites were highly conserved in bacteria. As shown in Figure 6C, 135 amino acid conserved sites were identified in *S. aureus*-A0A0E1VNV1 and *Bacillus cereus* (*B. cereus)*-A0A0J1KKV9 by site conservation analysis. The conserved amino acid sites accounted for 60% of the total amino acid sites. The results indicated that a large number

of amino acid sites identified between the two were homologous and highly conserved in both bacteria. Meanwhile, in the Kmal profiles of *S. aureus* and *B. cereus*, we found that the malonylation site K216 of *S. aureus*-A0A0E1VNV1 is more conserved than its counterpart in *B. cereus*. It was assumed that the malonylation site K216 is conserved in some bacteria. It was shown that more than 70% of the acetylated proteins are metabolic enzymes and translational regulators, suggesting that this modification is closely related to the regulation of energy metabolism and cellular function in *E. coli*. And it was found that a large number of direct homologs of lysine acetylation exist in *E. coli* and mammalian cells. So lysine acetylation may be prevalent in prokaryotic cells and the function of this modification may be evolutionarily conserved from bacteria to mammals (Zhang et al., 2009). Jennings et al. (2022) found that PTM alter the structure, function and localization of proteins and play key roles in physiological and pathological conditions. Many PTMs are derived from endogenous metabolic intermediates and act as sensors of metabolic feedback to maintain cell growth and homeostasis. Hardman-Kavanaugh et al. (2025) mention that when the environment changes rapidly, the cell cannot wait for newly synthesized proteins to respond to the challenge, and therefore achieves a rapid response by maintaining high expression levels of proteins and using PTM to regulate their activity. This mechanism allows cells to flexibly adjust protein functions to environmental changes without altering the genome. Therefore, malonylation modification of lysine and conserved malonylation sites may be involved in some metabolic regulation of bacteria affecting their growth or may be a beneficial adaptive trait acquired by bacteria during evolution. Colak et al. (2015) found that Kmal levels are closely related to MCD (malonic acid decarboxylase) activity. Kmal levels were significantly elevated in MCD-deficient mouse and human cells, suggesting that Kmal may play a key role in regulating fatty acid metabolism and energy supply. In prokaryotes, Kmal are involved in a variety of metabolic pathways, including the translation machinery, energy metabolism, RNA degradation and secondary metabolite biosynthesis. For example, in the cariogenic bacterium *Streptococcus mutans*, malonylated modified proteins are involved in biofilm formation and metabolic regulation (Li Z. et al., 2022). It can be seen that the presence of Kmal sites can affect various metabolisms, and metabolic regulation helps bacteria survive better under limited nutrition or environmental pressure. The evolutionary conservation of lysine malonylation modification in *S. aureus* were revealed, supporting its possible role in biofilm formation. It provides important clues for understanding the adaptive mechanism of bacteria and developing new antibacterial strategies. The current study was not validated by *in vitro* studies using malonylated proteins, and functional studies have not yet been completed. Future studies could further confirm the functionality of malonylation modification through *in vitro* studies and reveal the potential biofilm formation mechanism.

The down-regulation of AgrA expression observed in the quantitative proteome of *S. aureus* biofilm is likely to be an adaptive regulatory response of the bacterium to the biofilm microenvironment. This reaction involves complex regulatory mechanisms related to quorum sensing and may be regulated by PTMs, such as malonylation. Further studies are needed to clarify the role of AgrA in biofilm formation and maintenance and to explore the regulatory role of PTMs in AgrA function in biofilm micro environment.

### 3.8 Structure-activity analysis of AgrA and small molecule compounds

**FIGURE 7:**
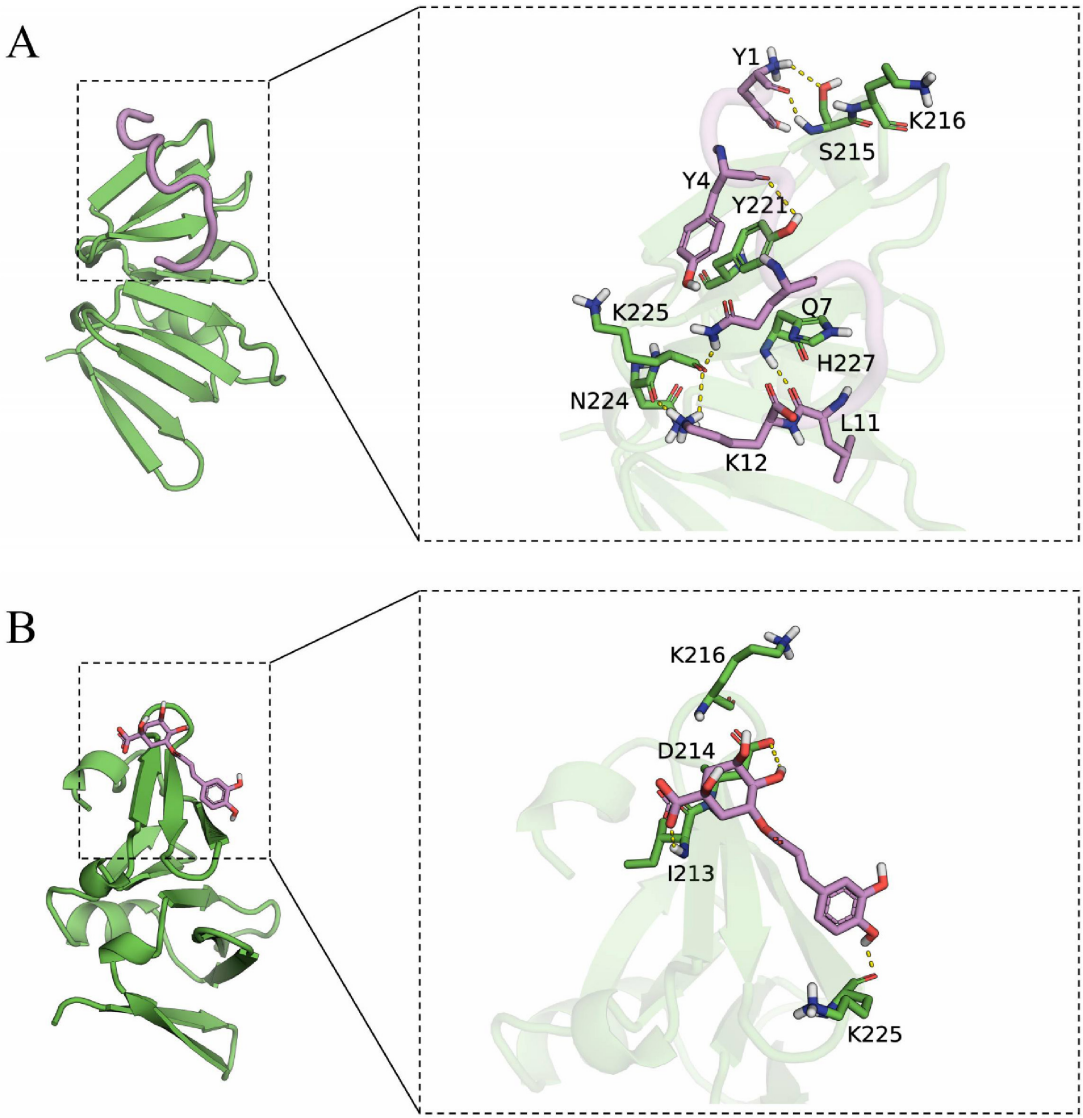
**(A)** Docking simulation between AgrA and BCp12; **(B)** Docking simulation between AgrA and CGA. The left image is the overall view, and the right image is the local view. The pink stick represents a small molecule, the green cartoon represents the protein, and the yellow dotted line indicates the hydrogen bond.

Docking simulation is a convenient and effective means to probe the interaction of small molecules with target targets. In this study, we performed docking studies of BCp12 and CGA with AgrA. Docking simulation of BCp12 and AgrA (Figure 7A) revealed that the Y1, Y4, Q7, L11, and K12 residues of the BCp12 form hydrogen bonds with the K216, S215, Y221, H227, N224, and K225 residues of the AgrA protein. Meanwhile, docking simulation of CGA and AgrA (Figure 7B) revealed that the CGA form hydrogen bonds with the D214, I213, and K225 residues of the AgrA protein.

In *S. aureus*, the antimicrobial peptide BCp12 inhibits bacterial growth and thus bacterial death by regulating Kmal levels in the arginine synthesis pathway, and also has an inhibitory effect on *S. aureus* biofilms (Shi et al., 2021). CGA at 5 mg/mL inhibited biofilm formation by 84.1% (Yu et al., 2024). Paul et al. (2021) demonstrated that AgrA can bind to tryptophan and inhibit *S. aureus* biofilm formation by interfering with the population-sensing system. Hydrogen bonds allow small molecules to bind with proteins. In molecular docking studies, a negative binding affinity indicates the possibility of binding, and smaller values indicate stronger binding. Our results showed that the binding affinity scores of BCp12 and CGA with AgrA were –6.888 and –5.302 kcal/mol, respectively, suggesting that both of them have the potential to bind to AgrA. Notably, molecular docking analyses revealed a stable hydrogen bonding network between BCp12 and the K216 and K225 sites and CGA and the K225 site of the AgrA protein, which are key Kmal sites in this protein. These findings indicate that BCp12 and CGA can target the Kmal modification sites of the AgrA protein, thereby inhibiting *S. aureus* DC15 biofilm formation.

## 4 Conclusion

Malonylation modification plays an important regulatory role in the formation of *S. aureus* biofilm. This study showed that the level of malonylation of proteins was significantly increased during biofilm formation. This modification participates in the formation of biofilms by affecting multiple key regulatory pathways including quorum sensing systems. Among them, AgrA protein showed a unique bimodal regulation mode: Its protein expression was significantly reduced, while the level of malonylation modification at some sites was correspondingly increased, suggesting that the malonylation modification of AgrA protein may have important regulatory functions in biofilm formation. Through molecular docking and functional characterization, we found that BCp12 and CGA can selectively bind to the malonylation site of AgrA. It shows that these modification sites have the potential as drug targets, which opens up a new way for antibacterial resistance through PTM-based strategies.

## Data Availability

The original contributions presented in the study are publicly available. This data can be found in the ProteomeXchange Consortium repository (https://proteomecentral.proteomexchange.org) with the accession numbers PXD066349 and PXD066374.

## Footnotes

1
http://www.genome.jp/kegg/

2
http://www.metaboanalyst.ca/
