# Supplementary material for: Identification of the malonylation modification in Staphylococcus aureus and insight into the regulators in biofilm formation
Source: Front Microbiol. 2025 Aug 19;16:1598098. doi: 10.3389/fmicb.2025.1598098 (PMC12404037; doi:10.3389/fmicb.2025.1598098)
Supplement: Supplementary file 3 [file Data_Sheet_3.pdf]

Figure. S1 Heatmap of the frequency distribution of amino acids around the Kmal sites of DMPs in *S. aureus*

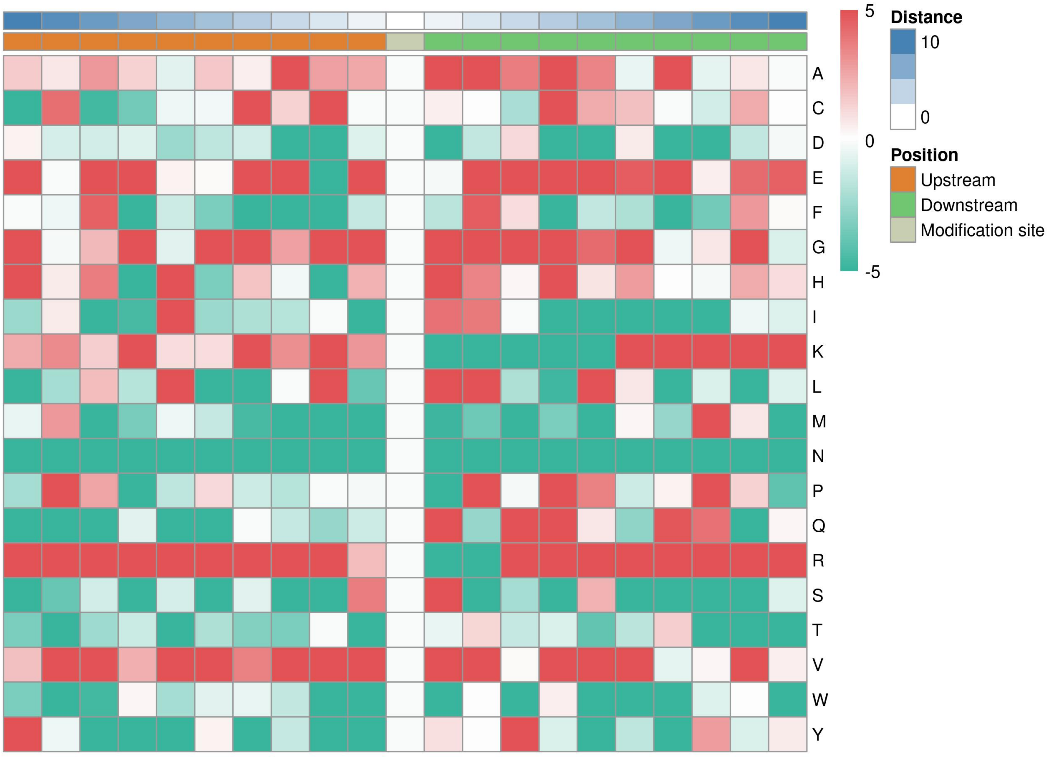

Figure. S2 Identification of Kmal motif; (E) Hierarchical cluster analysis of DMPs in *S. aureus* planktonic cells (0 h) and biofilm cells (96 h) ($P < 0.05$).

Motif Logo	Motif	Motif Score	Foreground		Background		Fold Increase
			Matches	Size	Matches	Size	
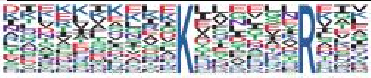	xxxxxxxxxx_K_xxxxxx Rxxx	15.83	241	3799	10715	297117	1.8
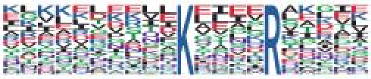	xxxxxxxxxx_K_xxxxRx xxxx	14.37	218	3558	9980	286402	1.8
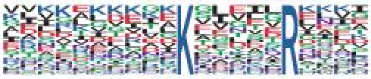	xxxxxxxxxx_K_xxxxxR xxxx	14.22	209	3340	9745	276422	1.8
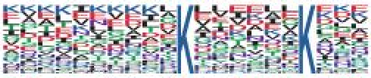	xxxxxxxxxx_K_xxxxxx Kxxx	10.22	430	3131	26904	266677	1.4
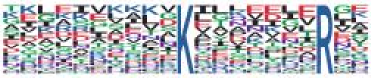	xxxxxxxxxx_K_xxxxxxx Rxx	11.42	159	2701	7855	239773	1.8
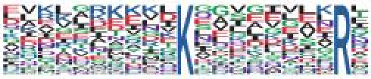	xxxxxxxxxx_K_xxxxxxx xRx	10.03	148	2542	7683	231918	1.8
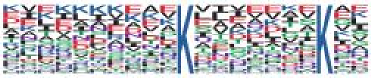	xxxxxxxxxx_K_xxxxxxx Kxx	8.44	330	2394	22510	224235	1.4
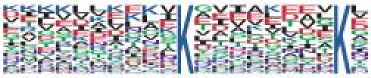	xxxxxxxxxx_K_xxxxxxx xKx	9.58	274	2064	18328	201725	1.5
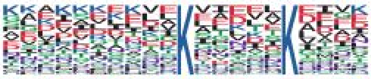	xxxxxxxxxx_K_xxxxxK xxxx	10.19	248	1790	16791	183397	1.5
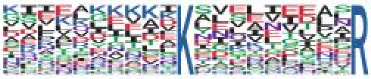	xxxxxxxxxx_K_xxxxxxx xxR	10.84	104	1542	5478	166606	2.1
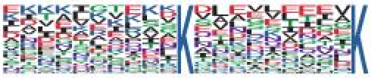	xxxxxxxxxx_K_xxxxxxx xxK	9.04	201	1438	14622	161128	1.5
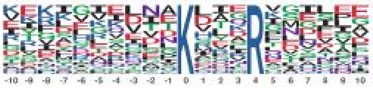	xxxxxxxxxx_K_xxxRxx xxxx	9.12	86	1237	4969	146506	2.0
